# Thrombophilic Disorders in Migraine

**DOI:** 10.3389/fneur.2014.00120

**Published:** 2014-07-10

**Authors:** Cinzia Cavestro, Silvia Mandrino

**Affiliations:** ^1^Headache Center, “S.Lazzaro” Hospital, Alba, Italy

**Keywords:** migraine, coagulative factors, protein S, protein C, anti-phospholipid, homocysteine

Migraine is a widespread disorder that affects about 18% of the people, in 2/3 of cases women (1).

The concept that migraine could be related to some vascular diseases, such as myocardial infarction or a stroke, have grown over the years, mainly in migraine with aura (2–6). In the last decades, several studies have been carried out to understand if and what the linkage could be. Among these studies, we have found many researches on thrombophilic disorders. In this context, the studies focus on alterations of coagulation factors, coagulation proteins, homocysteine with MTHFR variants, and auto-immune disorders of coagulation such as anti-phospholipid antibodies. The available data are sometimes difficult to interpret. The first problem we meet is that thrombophilic disorders usually have a low prevalence, so we need larger sample studies to reach significant results. The second problem could be how to correctly select the proper study population, because some deficits could be inherited and some others acquired, and results could change depending on contextual factors such as pregnancy or anti-coagulant or estroprogestinic therapy. The third problem, mainly dealing with auto-immune coagulative disorders, could be different laboratory methods and the different positivity criteria used. Another problem is that the prevalence of the coagulative deficit could change with age, so we find variable results in studying children or adults. Therefore, we need to calculate properly the sample size of the study population. Another chance to study the correlation between migraine and thrombophilic disorders could be by using the data collected through specific disease database. The main International Disease Database is the European Register on anti-phospholipid syndrome (APS). These data show that the first and most frequent symptom of the syndrome is a headache, probably a migraine considering the reported descriptions (7, 8). Headaches are so important in APS to lead expert hematologists to recommend carrying out tests for anti-phospholipid antibodies (aPL) in all patients presenting severe headaches (9). The Italian Registry of Rare Diseases is another important source of information. It was activated by the National Health Institute (NHI) almost 10 years ago, through the creation of Regional Registries. In the last Database Report of Rare Diseases’ 2013 database of the Piedmont Region, we find how the number of the diagnosis of a thrombophilic disease performed in an headache center is equal or higher than the one recorded by those centers taking care of patients who had experienced a thrombotic event (10). Articles published more recently correlate migraine and thrombophilic alterations, so we can presume they could be co-morbid.

Coagulative factors II, V, VII, VIII, IX, and von Willebrand have been studied in migraineurs. The correlation between factor II mutations and migraine has been studied throughout several smaller studies, always with negative results (11, 12). Only recently, a population based study reported a higher prevalence of factor II mutation in women suffering from migraine, but a selection bias could be suspected because this population had been selected among women with a positive family history of venous and arterial thrombosis (13). We can find a few reports of deficit of factor VII, which seems to be non-relevant (14, 15), and of factors von Willebrand, VIII e IX, and various platelet alloantigens (11, 16), topics that would be interesting to investigate further. Most authors studied the alterations of factor V in migraineurs, and also in these studies the sample size seem to be unrepresentative, and results are controversial (11, 15, 17–20). Furthermore, some authors considered homozygous mutation alone, while others considered both homozygous and heterozygous mutation. Among the coagulative factors, the V seems to be the most interesting one, but larger and better designed studies are required, with more severe selection of inclusion criteria.

Still little is known on deficit of coagulation proteins C and S. The main problem of studying protein C deficit is its low prevalence, which is around 0.4%, that requires larger sample sizes to study and detect any significant difference in prevalence between migraineurs and healthy controls. The few available studies are too small to clearly understand this topic (11, 18, 19). The prevalence of protein S deficit is higher, but this type of deficit is more complex to understand and diagnose. First of all, laboratory methods that are used should be clearly defined, such as the sensitivity of the laboratory kit used and what type of protein S was dosed, and is it the total or only the free protein, all elements needed to make a proper diagnosis of the type of defect (genetic or acquired). It would be better to distinguish inherited from acquired types of deficit, because of the different clinical relevance and etiopathogenesis. Inherited deficits are easier to detect and the level of protein S can be stable over time, on the contrary of the acquired deficits. It is also very important to consider and exclude iatrogenic deficits, such as those due to anti-coagulant or estroprogestinic therapy, or the physiologic lowering of protein S during pregnancy, in order to avoid misdiagnoses. Most difficult is dealing with auto-immune protein S deficits. In these cases, the level of protein S could change over time, depending on the level of auto-antibodies against protein S itself. The prevalence of acquired deficit of protein S is higher, this permits to study this type of deficit with lower sample size studies, but at least 100 people per group are required. In the literature, we can find very few and smaller studies, only one having sample size and selection criteria good enough to give reliable results (19). This study shows how migraineurs have a higher prevalence of protein S deficit. This data may be even more important in view of the correlation between estrogen, migraine, and stroke (21, 22).

In the last 20 years, the interest in the APS has grown more and more, and several articles have been published looking for any relationship between the syndrome and migraine. Dealing with this matter, we meet two types of problems: firstly, which criteria is the best to consider as a positive test; secondly, what type of laboratory test should be used, because of the high variability in sensibility and specifically they have. The diagnostic criteria accepted today are still those defined in the guidelines for the diagnosis of APS by Miyakis et al. (23, 24). Beyond the risk of thrombosis, the link between aPL and migraine seems supported by the two studies, which have fulfilled these criteria (25, 26). These studies confirm that the prevalence of aPL positivity in migraineurs is higher than in controls, both in children than in adults.

The interest in hyperhomocysteinemia or MTHFR variants is even higher than all the previous thrombophilic alterations. Several authors have been studying this topic, with smaller or larger populations, and variable results. For several years, hyperhomocysteinemia has been exclusively linked with MTHFR mutation, but nowadays this link does not seem to be so direct; in fact, hyperhomocysteinemia can be due to several other factors. Studying MTHFR variants seems to have a more speculative interest, and what could be the practical results of this knowledge is dubious. All these researches lead to uncertain significance of hyperhomocysteinemia, the results reporting both an increase of homocysteine in migraine with aura compared with healthy controls or no difference in larger population studies (27–30). Migraine with aura seems instead to be linked with the C677T variant of MTHFR gene (31–35). Isobe is the only researcher, who measured homocysteine level in cerebrospinal fluid, finding it higher in migraineurs, mainly with aura (36). Considering the previous reports of the bonding between migraine and hyperhomocysteinemia/MTHFR variants, some authors treated migraineurs with polivitaminic therapy, based on a complex of folic acid and various B vitamins, obtaining promising results (37, 38).

In conclusion, we consider thrombophilic coagulative alterations quite interesting for future studies, which should be very well designed, using rigid selection criteria and a correct sample size. Another very important aspect to be considered is to assess the impact that the therapies used to treat these diseases would have on migraine, especially considering that these therapies are easy to do and at low cost.

## Author Contributions

Cinzia Cavestro wrote the final report. Mrs. Silvia Mandrino co-wrote the final report.

## Conflict of Interest Statement

The authors declare that the research was conducted in the absence of any commercial or financial relationships that could be construed as a potential conflict of interest.
